# Supplementary figures and images for: Transgenic mice overexpressing Pitx2 in the atria develop tachycardia-bradycardia syndrome
Source: PLoS One. 2025 Sep 4;20(9):e0330397. doi: 10.1371/journal.pone.0330397 (PMC12410714; doi:10.1371/journal.pone.0330397)

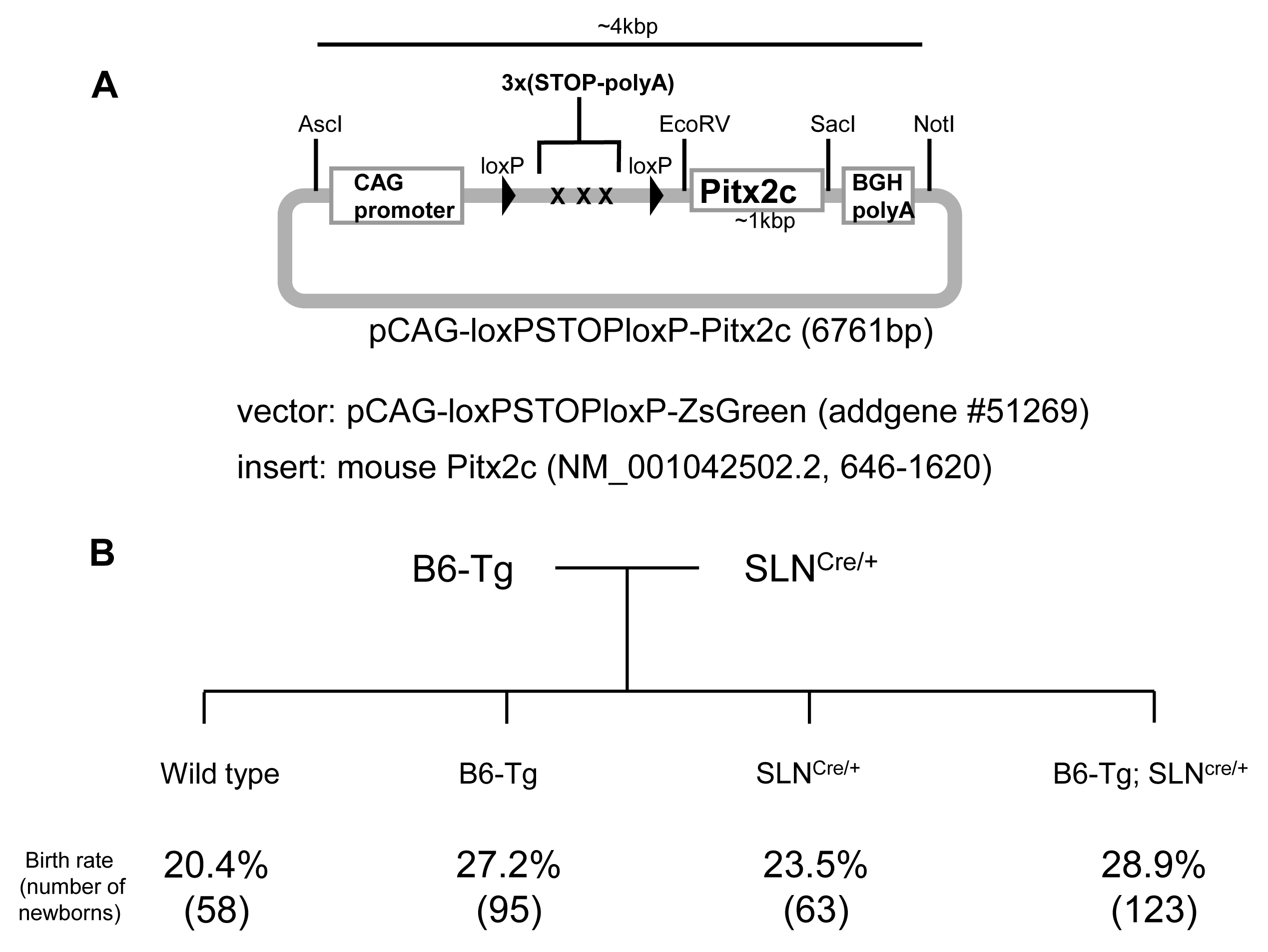

Supplement: S1 Fig — A, We generated the genetic construct for the conditional overexpression of Pitx2c mice. B, We obtained four types of mice, which were born at more than the expected Mendelian ratio. (TIF) [file pone.0330397.s001.tif]

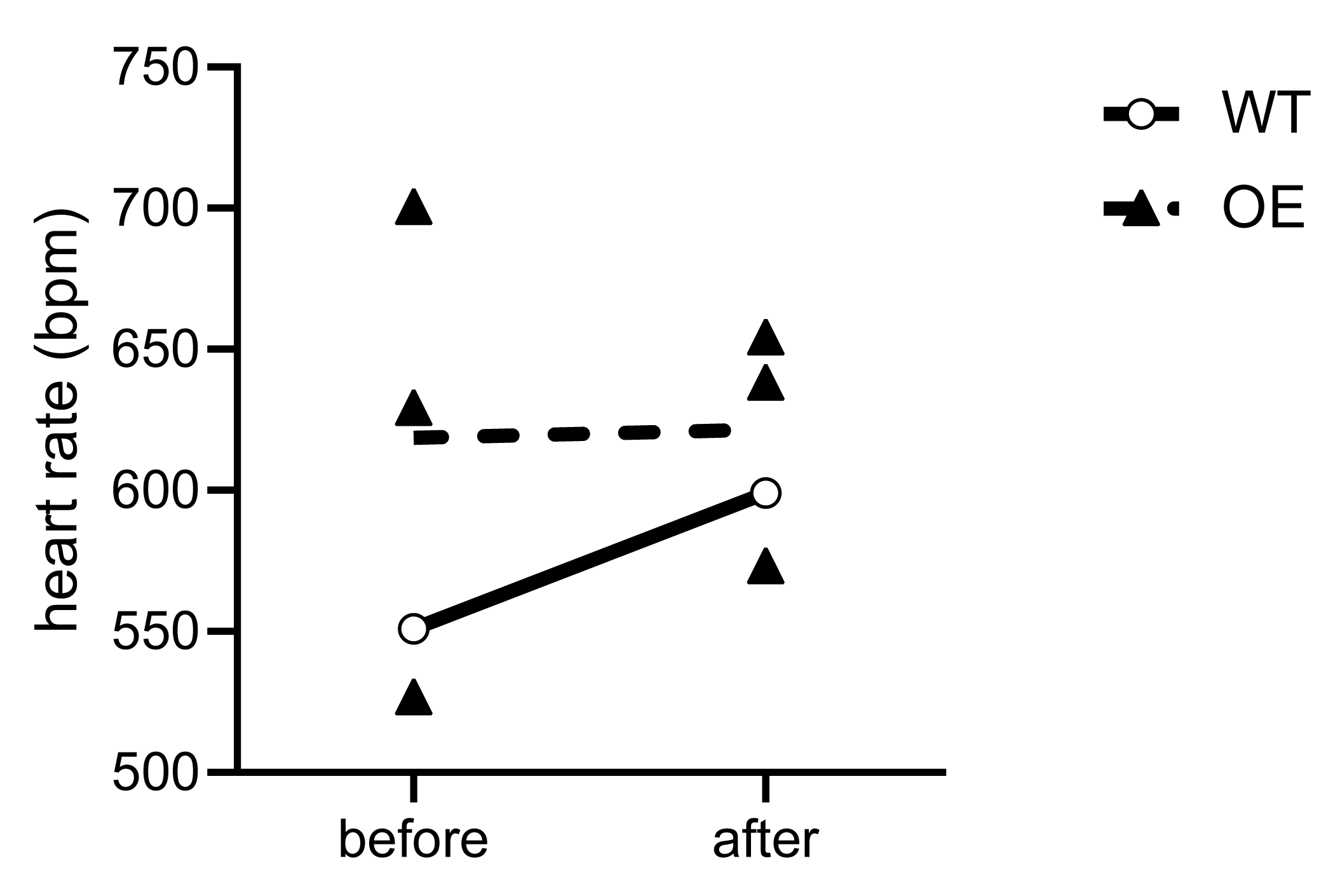

Supplement: S2 Fig — This figure shows that heart rate of WT and OE mice before and after pharmacological blockade of the autonomic nervous system. White circle and solid line show WT mice, and black tringle and dotted line show OE mice. (n = 3 per group). (TIF) [file pone.0330397.s002.tif]

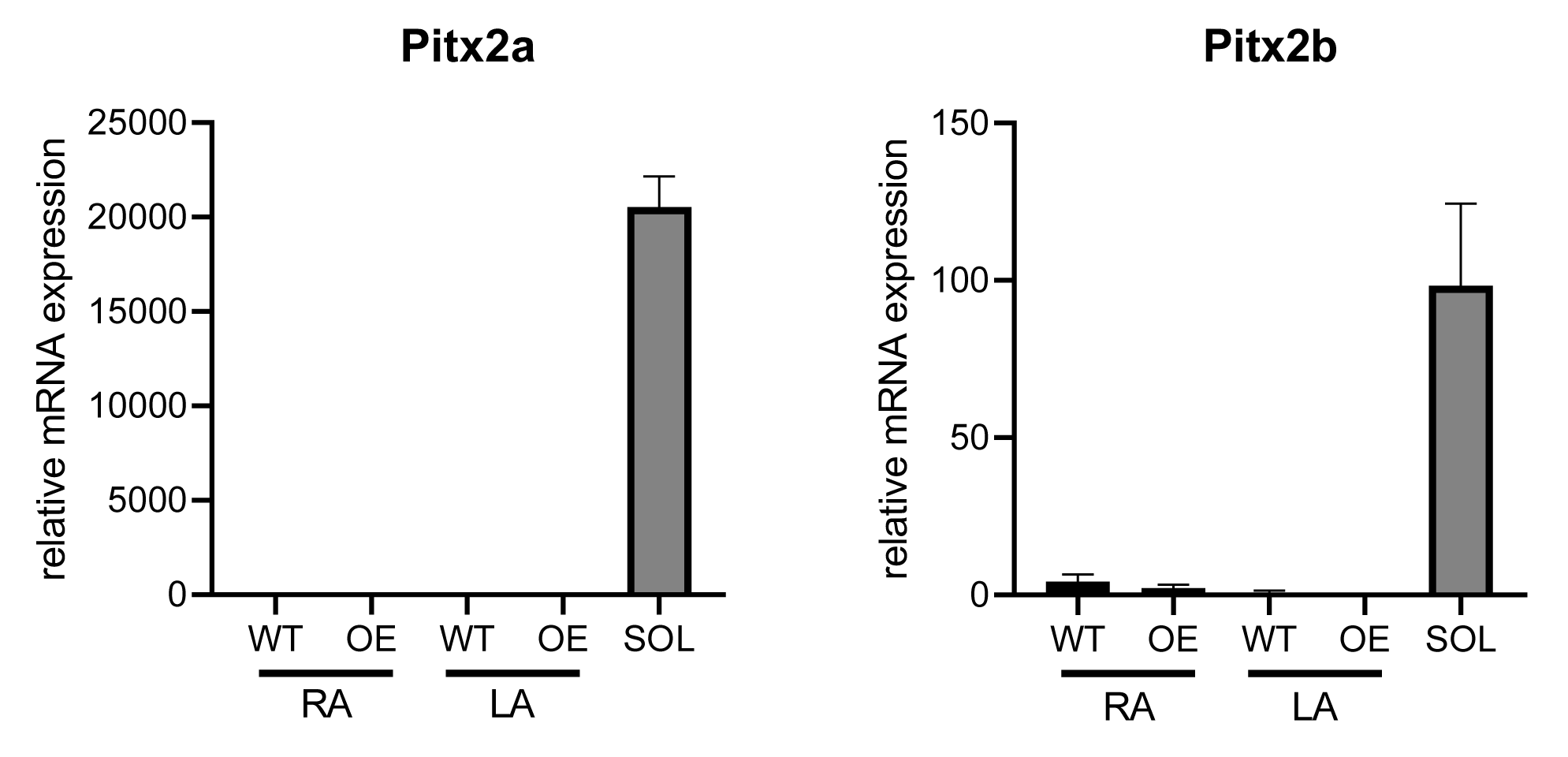

Supplement: S3 Fig — RTPCR shows that the Pitx2a and Pitx2b isoform in the mouse heart is almost less expressed than the soleus as the positive control. The data are the mean ± standard error of the mean (n = 4 per group, #p < 0.05). (TIF) [file pone.0330397.s003.tif]

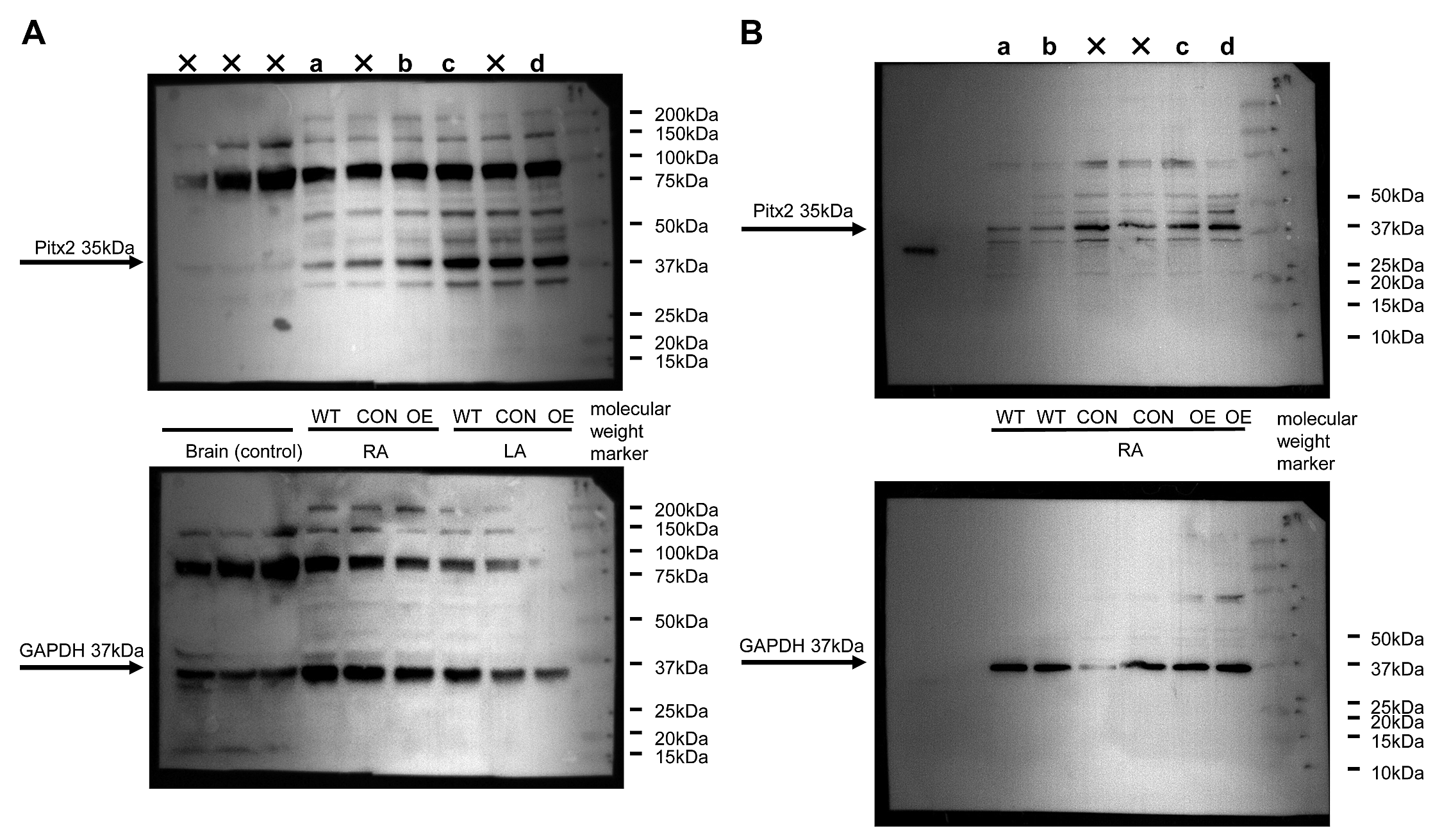

Supplement: S4 Fig — A, The protein of Pitx2 increased in the RA of OE mouse compared with that in the RA of WT mouse. a lane: RA of WT mouse, b lane: RA of OE mouse, c lane: LA of WT mouse, d lane: LA of OE mouse, WT: wild type mouse, CON: B6-Tg (CAG-LSL-Pitx2c) mouse, OE: B6-Tg (CAG-LSL-Pitx2c); SLNCre/ + mouse, B, The protein of Pitx2 increased clearly in the RA of OE mouse compared with that in the RA of WT mouse. a and b lane: RA of WT mouse, c and d lane: RA of OE mouse, WT: wild type mouse, CON: B6-Tg (CAG-LSL-Pitx2c) mouse, OE: B6-Tg (CAG-LSL-Pitx2c); SLNCre/+ mouse. (TIF) [file pone.0330397.s004.tif]

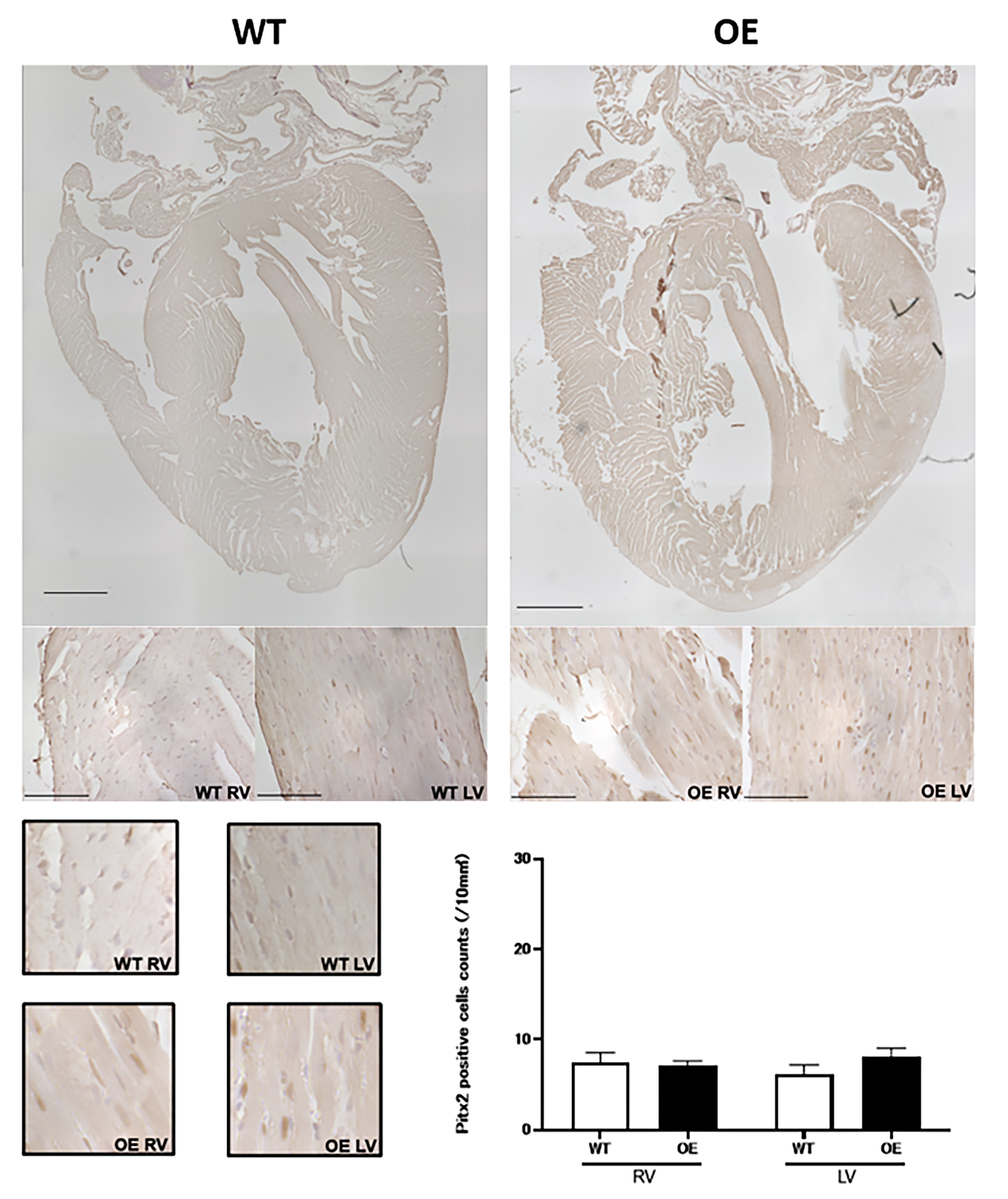

Supplement: S5 Fig — Upper figures didn’t show the expression of Pitx2 clearly (scale bar = 1000μm). Middle figures, lower left and right figures showed the weak and similar expression of Pitx2 in the both ventricles in WT mouse and OE mouse (scale bar = 100μm). (TIF) [file pone.0330397.s005.tif]

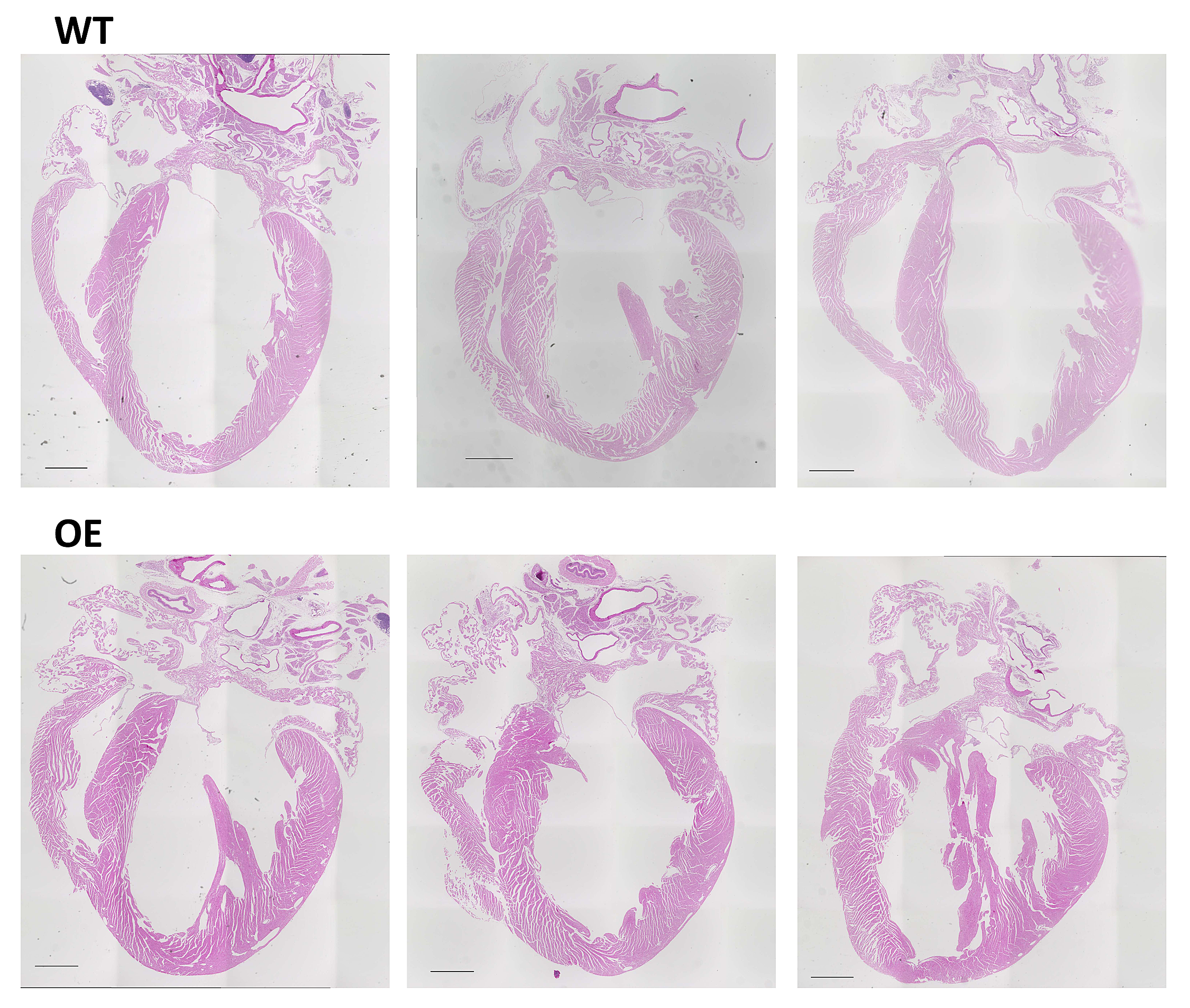

Supplement: S6 Fig — These figures of HE staining of the whole heart show no difference in morphology between WT and OE mice (scale bar = 1000μm). (TIF) [file pone.0330397.s006.tif]

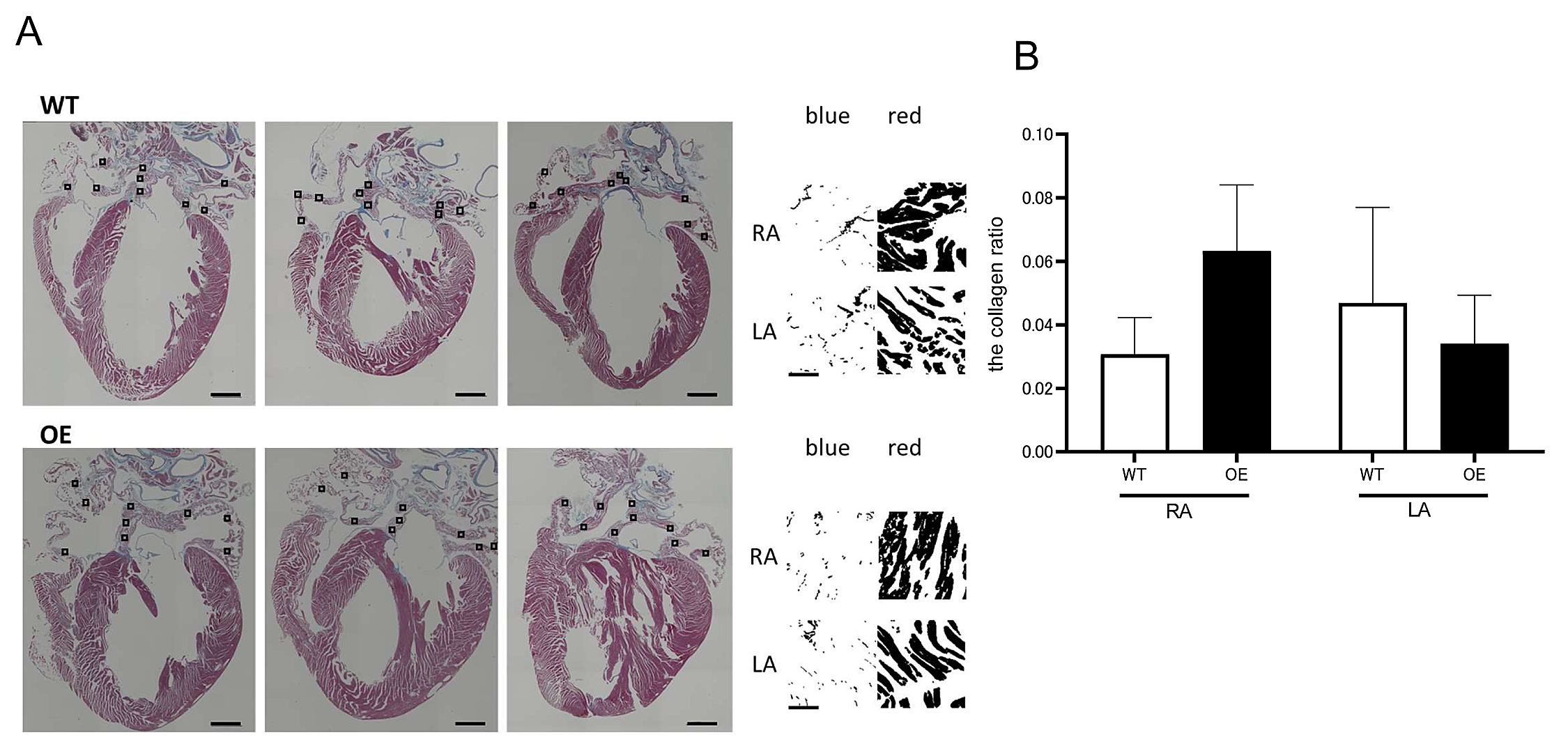

Supplement: S7 Fig — A, These figures of MT staining of the whole heart show no difference in morphology (scale bar = 1000μm). And, fibrosis was assessed with Masson’s trichrome staining. using ImageJ (red: myocytes, blue: collagen fibers) from dotted squares of three images randomly selected in the RA and LA from MT-stained hearts of WT and OE mice (scale bar = 10μm). B, The collagen fiber ratio was defined as the area of collagen fibers divided by the area of collagen fibers and myocytes). No significant difference in fibrosis was observed between RA and LA of WT and OE mice. (TIF) [file pone.0330397.s007.tif]

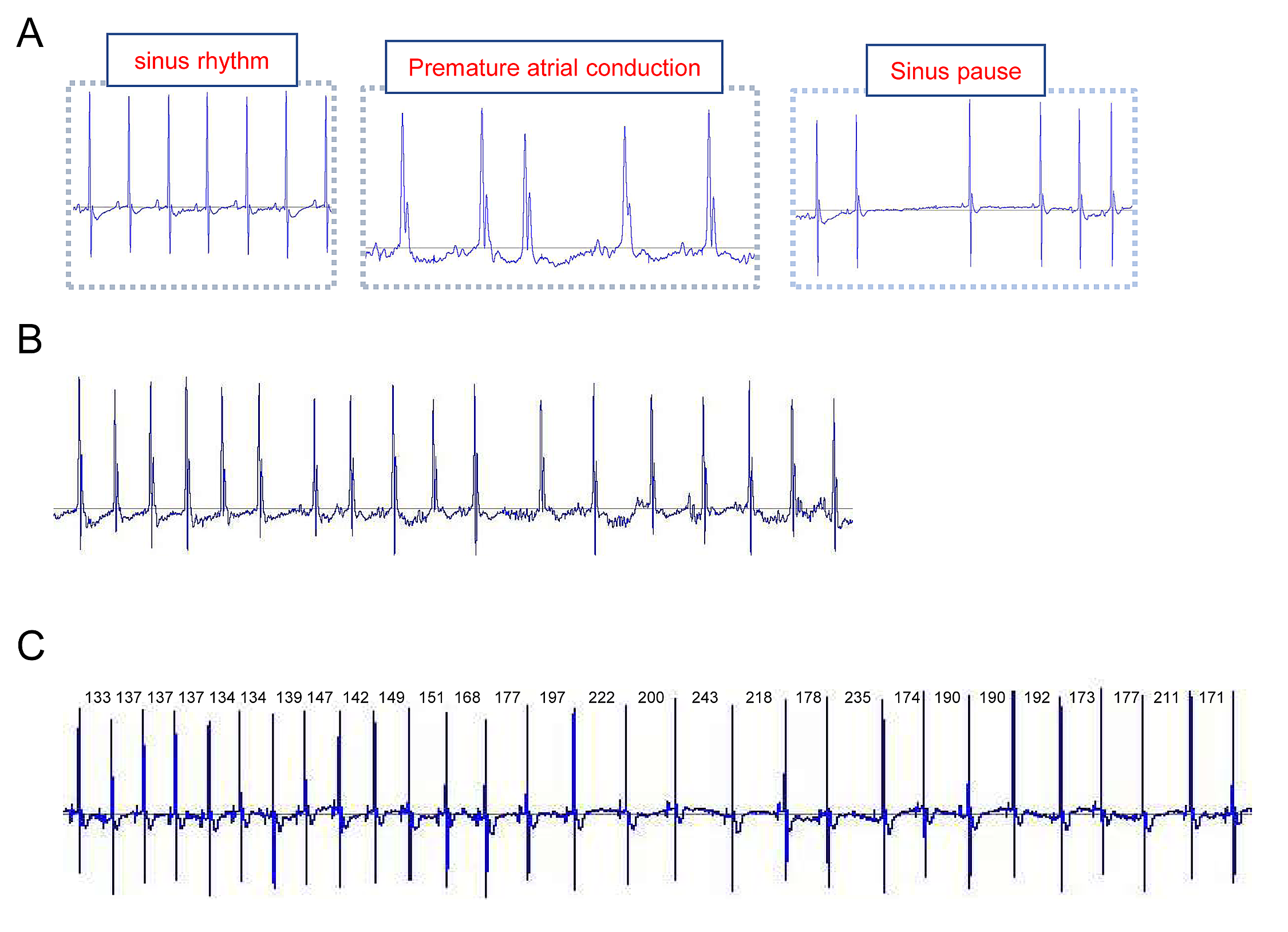

Supplement: S8 Fig — A, The telemetric data show representative examples of ECG waves for sinus rhythm and arrhythmias. B and C, The telemetric data recorded the waves of AF and tachycardia-bradycardia. The number above the wave shows each RR interval length. (TIF) [file pone.0330397.s008.tif]

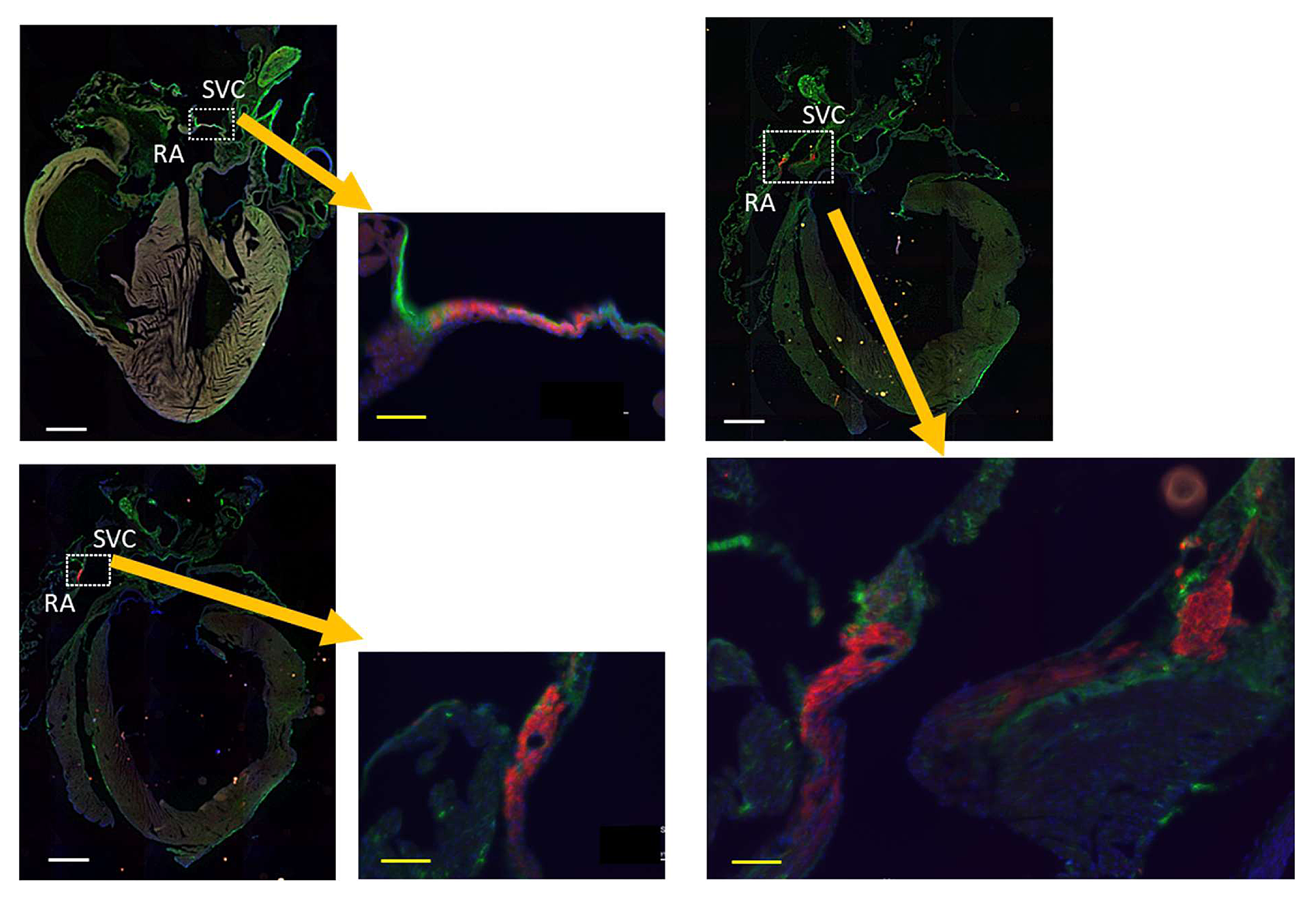

Supplement: S9 Fig — Three representative fluorescence immunostaining of the whole heart and the SVC-RA junction in WT mice. There was a clear cluster of HCN4-positive cells, which characterizes the location of the sinus node, at the SVC-RA junction of WT mice. (TIF) [file pone.0330397.s009.tif]

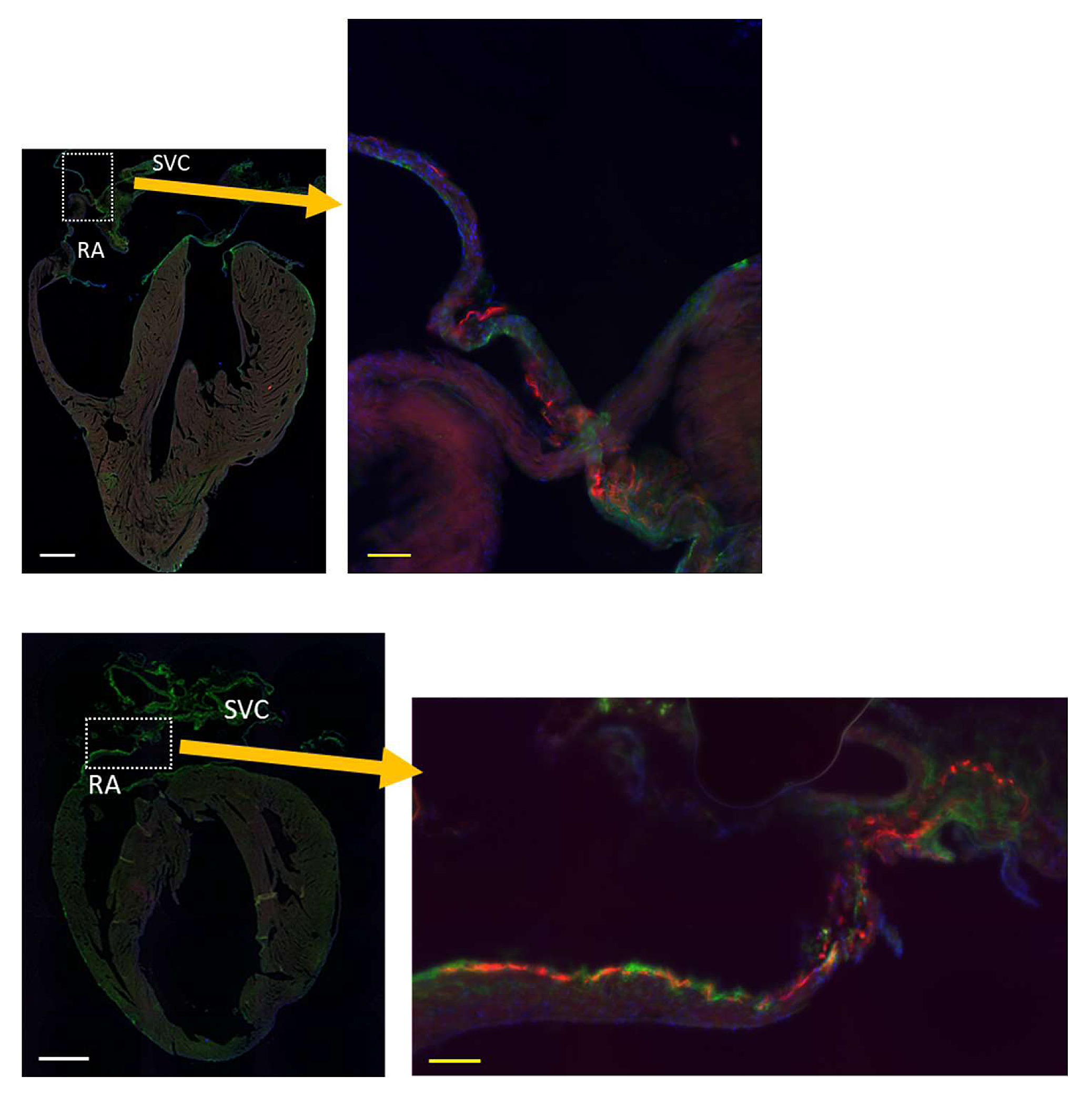

Supplement: S10 Fig — Three representative fluorescence immunostaining of the whole heart and the SVC-RA junction in OE mice. HCN4-positive cells were a widespread or ectopic presence, whereas the clustering of HCN4-positive cells at the SVC-RA junction was rare or absent. (ZIP) [file pone.0330397.s010.zip › S10 Fig-1 revision.tif]

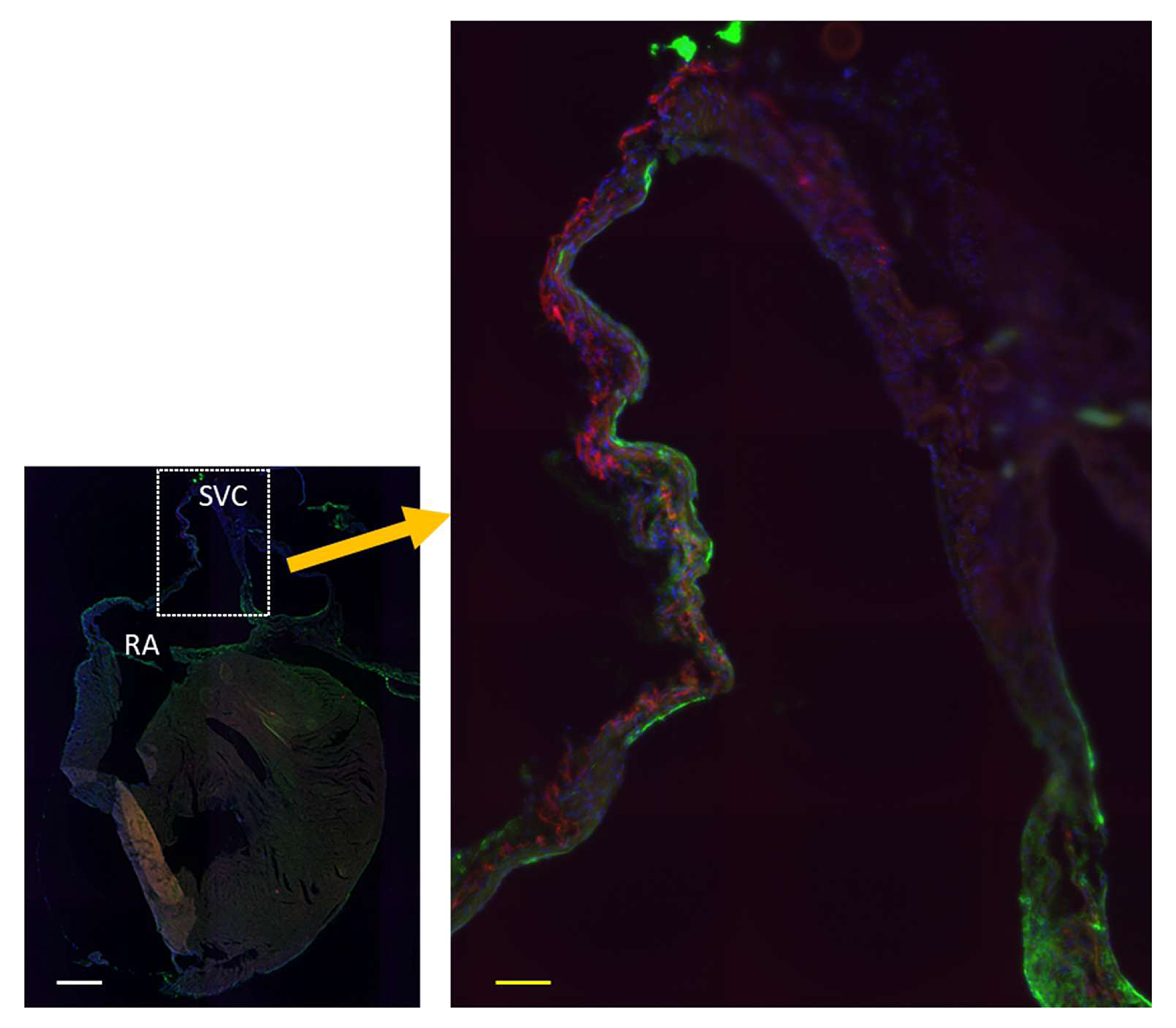

Supplement: S10 Fig — Three representative fluorescence immunostaining of the whole heart and the SVC-RA junction in OE mice. HCN4-positive cells were a widespread or ectopic presence, whereas the clustering of HCN4-positive cells at the SVC-RA junction was rare or absent. (ZIP) [file pone.0330397.s010.zip › S10 Fig-2 revision.tif]

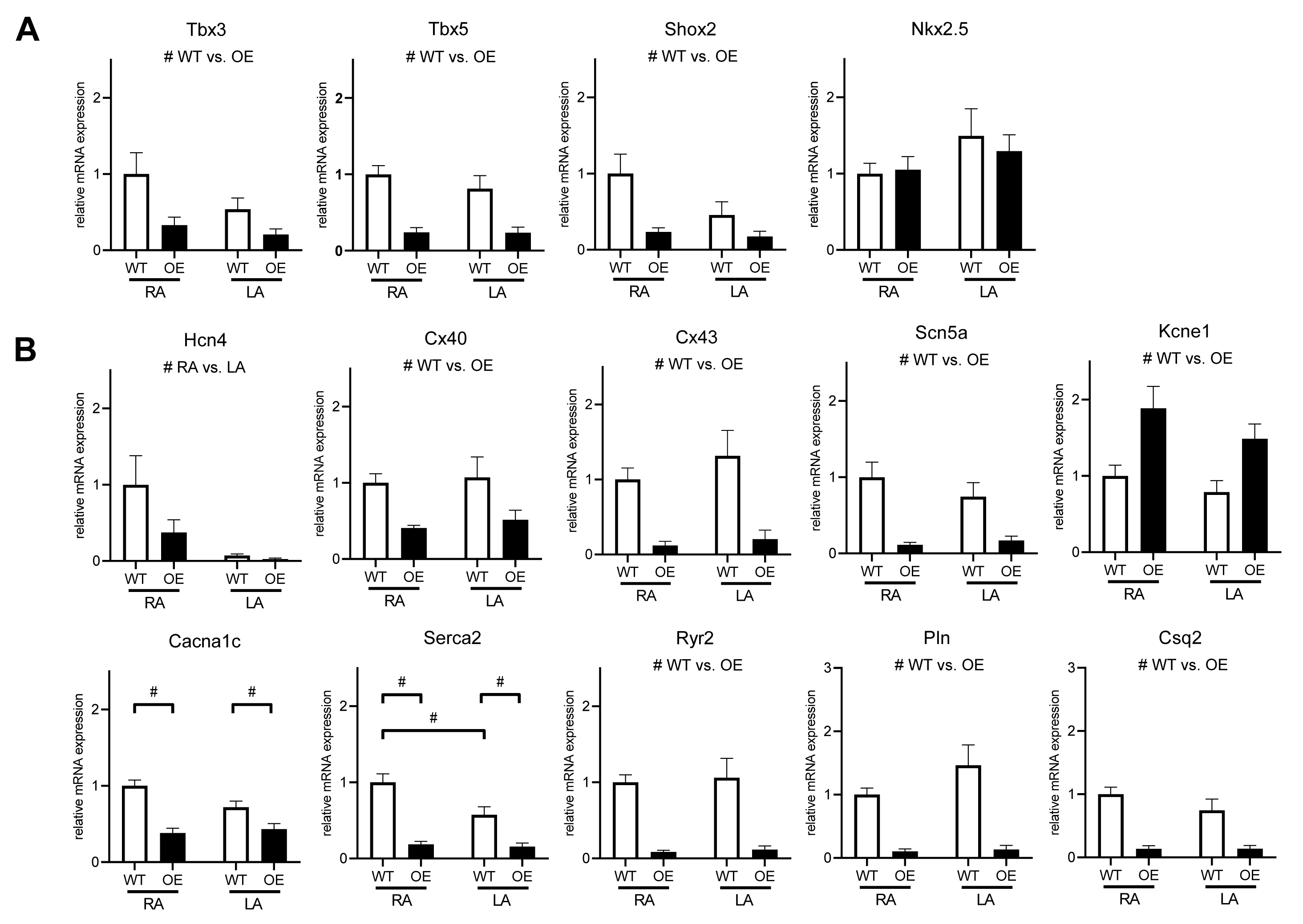

Supplement: S11 Fig — In OE mice, mRNAs were significantly changed. In particular, mRNAs (Cacna1c, Serca2) related to Ca handling which promoted AF were reduced in the RA of OE mice. The data are the mean ± standard error of the mean (n = 8 per group, #p < 0.05). (TIF) [file pone.0330397.s011.tif]

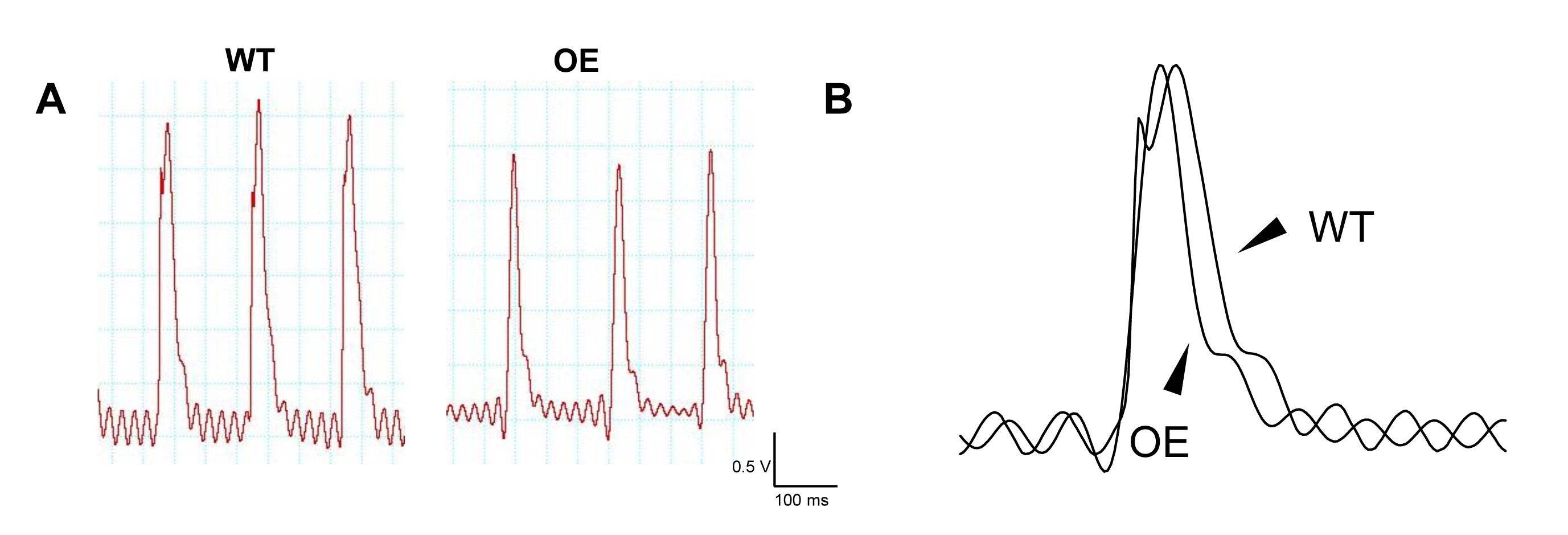

Supplement: S12 Fig — A, Atrial MAP was recorded without pacing and with pacing by 8 Hz, 10 Hz, and 12 Hz. B, Overlay of recordings in the MAP about RA of OE mice and RA of WT mice. (TIF) [file pone.0330397.s012.tif]

**A**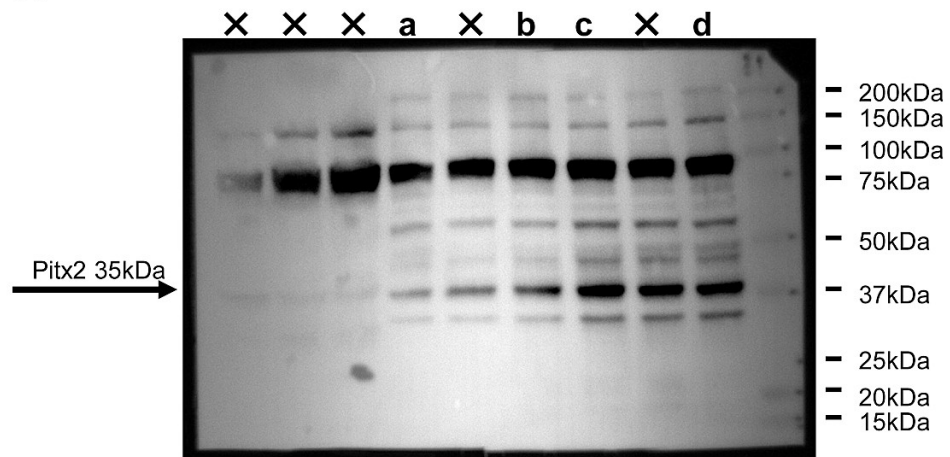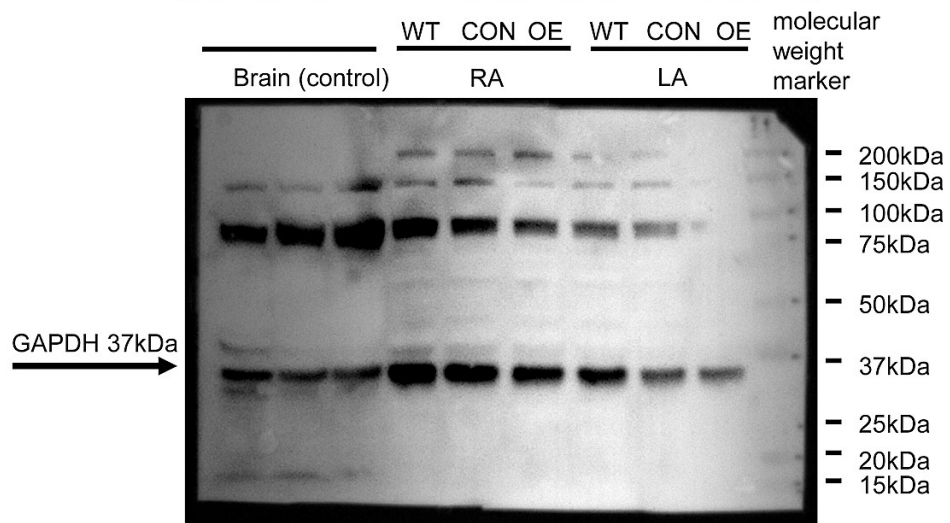**B**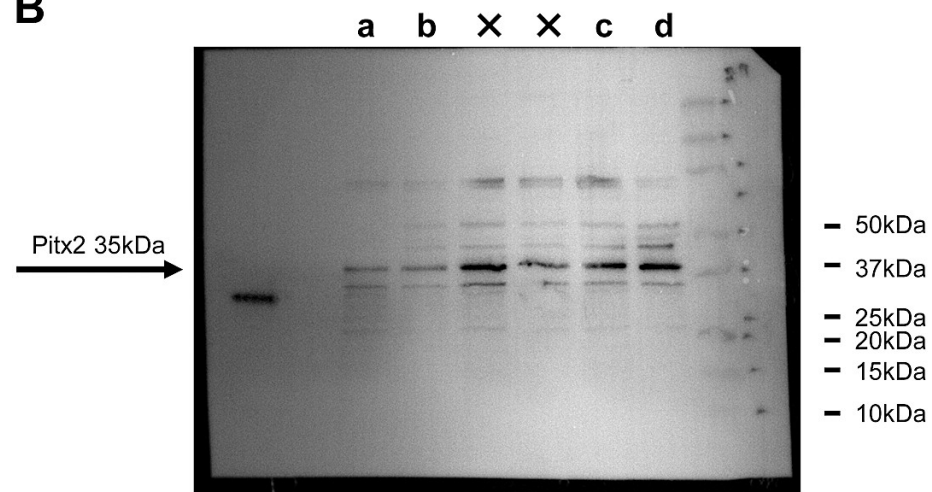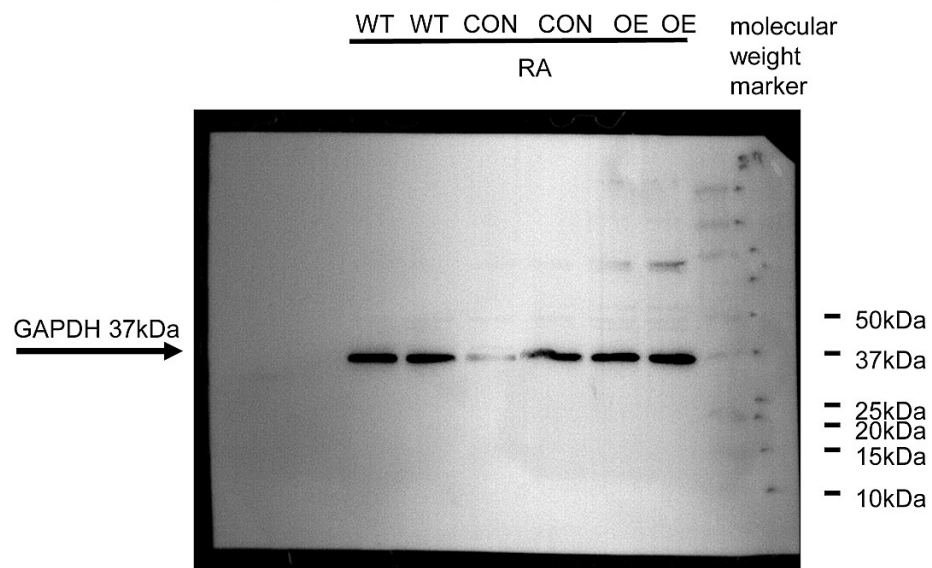

Supplement: S1 File — (PDF) [file pone.0330397.s017.pdf]
