# Supplementary material for: Transgenic mice overexpressing Pitx2 in the atria develop tachycardia-bradycardia syndrome
Source: PLoS One. 2025 Sep 4;20(9):e0330397. doi: 10.1371/journal.pone.0330397 (PMC12410714; doi:10.1371/journal.pone.0330397)
Supplement: S1 Table — Primers which were used for polymerase chain reaction screening of the DNA. (DOCX) [file pone.0330397.s013.docx]

S1 Table.

| mouse qRT-PCR | Oligonucleotide sequence |
| --- | --- |
| Cre Forward | 5’- acgggaagggttggcaagtca -3’ |
| Cre Reverse | 3’- tcctcttcttcttgggcatgg -5’ |
| Pitx2c Forward | 5’-ctctagagcctctgctaaccatgt-3’ |
| Pitx2c Reverse | 5’-ccagcagccaacgcacccat-3’ |
| Pitx2a Forward | 5’- gcctgcgtgcaattagagaaag -3’ |
| Pitx2a Reverse | 3’- tgctggctagtgaaatgagtcc -5’ |
| Pitx2b Forward | 5’- aaaggtcgagttcacggactc -3’ |
| Pitx2b Reverse | 3’- agcggtttctctggaaagtgg -5’ |
| Gapdh Forward | 5’- tcctgcaccaccaactgcttag -3’ |
| Gapdh Reverse | 3’- gatgaccttgcccacagccttg -5’ |
| Tbx3 Forward | 5’- agagatggtcatcacgaagtcagg -3’ |
| Tbx3 Reverse | 3’- cgacagtcgtcagcagctataatg -5’ |
| Tbx5 Forward | 5’- acgaagtgggcacagagatg -3’ |
| Tbx5 Reverse | 3’- cgtctgcgggaacaatatccatg -5’ |
| Shox2 Forward | 5’- gctttcatgcgcgaggaattg -3’ |
| Shox2 Reverse | 3’- acgttgacatagggtgcaactc -5’ |
| Cx40 Forward | 5’- tcatctttgtgtccacgccttc -3’ |
| Cx40 Reverse | 3’- tctcagcatcccgcaatttctg -5’ |
| Cx43 Forward | 5’- gtacccaacagcagcagactttg -3’ |
| Cx43 Reverse | 3’- tggagtaggcttggaccttgtc -5’ |
| Hcn4 Forward | 5’- aaagagaccaagctggctgatg -3’ |
| Hcn4 Reverse | 3’- gcacctcattgaagttgtccac -5’ |
| Scn5a Forward | 5’- gctcatcatgtgcaccatcctaac -3’ |
| Scn5a Reverse | 3’- aggaaggtgaacgcatgcag -5’ |
| Nkx2.5 Forward | 5’- cttcaagcaacagcggtacct -3’ |
| Nkx2.5 Reverse | 3’- cgctgtcgcttgcacttgta -5’ |
| Kcne1 Forward | 5’- tcctagtgaatgtcgccttgtttg -3’ |
| Kcne1 Reverse | 3’- ctctcgtggcactctacggt -5’ |
| Cacna1c Forward | 5’- ggagcaagggttcaatatcacca -3’ |
| Cacna1c Reverse | 3’- gaaagctcaaaccaactcgtagtg -5’ |
| Serca2 Forward | 5’- tggaacaacccggtaaagagt -3’ |
| Serca2 Reverse | 3’- caccaggggcataatgagcag -5’ |
| Ryr2 Forward | 5’- cgtagtaacaaagctgtgggctg -3’ |
| Ryr2 Reverse | 3’- agtcagtaaccaattcctgcgg -5’ |
| Phospholamban Forward | 5’- tgtgtagttggccatgctgagag -3’ |
| Phospholamban Reverse | 3’- gtggtccgtaaacaatataacgagt -5’ |
| Csq2 Forward | 5’- atttatggatgagcccaacg -3’ |
| Csq2 Reverse | 3’- gtcactcttctccgcaaagg -5’ |

Primers which were used for polymerase chain reaction screening of the DNA.
