# Supplementary material for: Transgenic mice overexpressing Pitx2 in the atria develop tachycardia-bradycardia syndrome
Source: PLoS One. 2025 Sep 4;20(9):e0330397. doi: 10.1371/journal.pone.0330397 (PMC12410714; doi:10.1371/journal.pone.0330397)
Supplement: S2 Table — The table shows the parameters for the ratio of atrial and ventricular weights to body weight in each group at 10–13 weeks, echocardiographic and electrocardiac evaluation at 8 weeks. The data are the mean ± standard error of the mean (n = 8 per group, but n = 7 Pwave height and duration). (DOCX) [file pone.0330397.s014.docx]

S2 Table. Parameters of each group

|  | **WT** | **OE** |
| --- | --- | --- |
| Age, days | 102.5±5.30 | 105.0±5.20 |
| BW, mg | 26.4±1.80 | 23.9±1.80 |
| LA/BW, ×10^-2^ | 1.53±0.10 | 1.25±0.04 |
| RA/BW, ×10^-2^ | 1.78±0.13 | 1.56±0.12 |
| LV/BW, ×10^-2^ | 34.2±1.19 | 41.0±0.04 |
| RV/BW, ×10^-2^ | 10.7±0.66 | 11.5±1.15 |
| **echocardiography** |  |  |
| HR, bpm | 433±7.18 | 407±23.9 |
| LVPWs, mm | 1.02±0.06 | 0.84±0.05 |
| LVPWd, mm | 0.76±0.05 | 0.64±0.08 |
| LVIDs, mm | 2.73±0.17 | 2.91±0.12 |
| LVIDd, mm | 3.81±0.16 | 3.95±0.11 |
| IVSs, mm | 1.05±0.08 | 0.94±0.04 |
| IVSd, mm | 0.65±0.09 | 0.68±0.05 |
| FS, % | 28.4±2.46 | 26.6±1.39 |
| P valve, mm | 1.41±0.07 | 1.38±0.06 |
| RA a, mm^2^ | 2.89±0.04 | 2.53±0.23 |
| LA a, mm^2^ | 2.38±0.13 | 2.20±0.20 |
| RVEDV, mm^2^ | 8.61±0.27 | 10.3±0.46 |
| LVEDV, mm^2^ | 8.53±0.23 | 10.6±0.45 |
| PA VTI, mm | 21.0±2.31 | 18.7±1.30 |
| CO, ml/min | 13.9±1.65 | 11.3±1.22 |
| PAT, ms | 17.6±1.08 | 18.7±1.28 |
| PET, ms | 61.6±2.36 | 67.1±2.85 |
| PAT/PET | 0.29±0.02 | 0.28±0.02 |
| **electrocardiography** |  |  |
| PR, msec | 34.8±0.91 | 34.0±1.41 |
| QRS, msec | 13.1±0.20 | 13.2±0.53 |
| QTc, msec | 40.2±0.67 | 43.3±1.45 |
| Pwave height, mV | 0.19±0.020 | 0.16±0.021 |
| Pwave duration, msec | 11.2±0.080 | 10.4±0.076 |

The table shows the parameters of atrial and ventricular weights at 10 to 13 weeks, echocardiographic and electrocardiac evaluation at 8 weeks in each group. The data are the mean ± standard error of the mean (n = 8 per group, but n=7 Pwave height and duration).
