# Supplementary material for: Transgenic mice overexpressing Pitx2 in the atria develop tachycardia-bradycardia syndrome
Source: PLoS One. 2025 Sep 4;20(9):e0330397. doi: 10.1371/journal.pone.0330397 (PMC12410714; doi:10.1371/journal.pone.0330397)
Supplement: S3 Table — The data are the mean ± standard error of the mean (n = 8 per group). max SNRT: maximum sinus node recovery time of three trials; duration and amplitude: the duration and amplitude of the stimulation for 30 s. Total experimental duration: the experimental time from the anesthesia to the end. (DOCX) [file pone.0330397.s015.docx]

S3 Table.

|  | WT | OE |
| --- | --- | --- |
| max SNRT, ms | 358±36.9 | 437±32.4 |
| BHR, bpm | 280±14.6 | 313±8.10 |
| Cycle length, ms | 162±4.00 | 156±2.77 |
| amplitude, mV | 0.108±0.0117 | 0.11±0.0152 |
| total experimental duration, min | 31±3.1 | 32±1.8 |

Parameters for the overdrive suppression test. The data are the mean ± standard error of the mean (n =6, 9, per group).

max SNRT: maximum sinus node recovery time of three trials.

duration and amplitude: the duration and amplitude of the stimulation for 30 sec.

Total experimental duration: the experimental time from the anesthesia to the end.
